# Warehouse Fire Detection System Based on Multi-Sensor Information Fusion

**DOI:** 10.3390/s26123763

**Published:** 2026-06-12

**Authors:** Ziqiang Zhang, Yuxuan Ye, Xiaodong Wang, Xinqi Zhi, Xinpeng Zhang, Mingxing Zhang

**Affiliations:** 1 Engineering Training Center, Changchun University of Technology, Changchun 130012, China; 2 School of Mechanical and Electrical Engineering, Changchun University of Technology, Changchun 130012, China; 3 Research Laboratory of Logging, Material Preparation and Fire Prevention & Control Technology and Equipment, Harbin Research Institute of Forestry Machinery, State Forestry and Grassland Administration, Harbin 150086, China

**Keywords:** fire detection, multi-sensor information fusion, particle swarm optimization algorithm, BP neural network

## Abstract

To address the problems of false negatives, false positives, and delayed response in traditional fire detection systems, this paper proposes a warehouse fire detection scheme based on multi-sensor information fusion. By constructing a ZigBee wireless sensor network and integrating temperature, CO concentration and smoke sensors, fire simulation data are collected in the warehouse. At the data processing level, an improved Grubbs criterion is innovatively adopted to eliminate outliers, and the median is used instead of the average to effectively suppress the same-side shielding effect. At the feature layer fusion stage, a BP neural network model optimized by the cosine decreasing inertia weight particle swarm optimization algorithm (CIW-PSO) is designed. By dynamically adjusting the learning factors (c_1_, c_2_) and inertia weight (w), the convergence speed and global optimization ability are significantly improved. At the decision-making level, a fuzzy logic reasoning mechanism is introduced to integrate multi-parameter membership functions, thereby reducing the probability of misjudgment. Field tests have verified that the system can achieve early fire warning in a 50 m × 100 m warehouse environment, with a false alarm rate reduced by 42% compared to a single sensor and a response time shortened by 35%, providing an efficient and reliable intelligent solution for warehouse fire safety.

## 1. Introduction

As the economy develops, the market size of China’s warehousing industry has continued to expand. According to data from the China Research and Analysis Industry Research Institute, the China Business Industry Research Institute, and other organizations, the market size of China’s intelligent warehousing industry (including integration business and software business) has increased from 88.29 billion yuan in 2019 to 153.35 billion yuan in 2023, with an average annual compound growth rate of 14.80%. In 2024, the scale of China’s intelligent warehousing industry further grew to 176.05 billion yuan [1]. Fire accidents frequently occur in daily life and production, especially in facilities like warehouses; these incidents can often result in significant losses. Therefore, it is essential to develop a more intelligent, efficient, and reliable digital fire detection system.

The primary advantage of a multi-sensor fire detection system over a single detector lies in its ability to comprehensively analyze multiple characteristic parameters, thereby reducing the likelihood of false alarms. Consequently, the selection of appropriate fire-related parameters becomes critically important. During a fire, numerous physical and chemical reactions occur, causing significant changes in surrounding environmental conditions; detecting these changes enables the identification of a fire incident. Fire combustion produces two major categories of products: gases and solids. In the open flame phase, fires release substantial heat, leading to a marked increase in ambient temperature—one of the most distinctive characteristics of any fire. The combustion products of gases mainly include CO, CO_2_, and H_2_, among which CO and CO_2_ are produced in larger quantities. Under normal circumstances, the concentrations of these two gases in the air are very low, approximately 8 ppm and 320 ppm respectively. However, due to the production of CO_2_ from biological respiration, in relatively closed and poorly ventilated environments, detectors may cause false alarms due to excessively high local CO_2_ concentrations. Additionally, during a fire, the concentration of CO in the environment can rise to several hundred or even thousands of ppm, showing a very obvious change. Therefore, CO is more suitable than CO_2_ as a fire characteristic parameter. The combustion products of solids mainly consist of incompletely burned liquid and solid decomposition products, such as smoke particles visible to the naked eye with diameters ranging from 0.01 to 10 μm and some unburned solid particles. Smoke is generated in all stages of a fire, making it another important characteristic of a fire occurrence [2].

**Figure 1:**
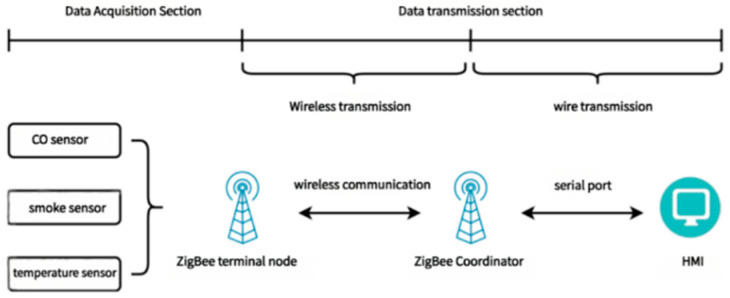
Hardware structure of the multi-sensor fire detection system.

As shown in Figure 1, this paper selects temperature, CO, and smoke sensors. To address the complex wiring and high-cost issues of current bus-type fire detectors in the market, ZigBee wireless communication technology is used for connection [3,4].

ZigBee is a wireless communication standard formulated by ZigBee Alliance [5], which has the characteristics of low delay, low power consumption and large network capacity, and has strong ad hoc networking capability, and ZigBee protocol is free, the use cost is low, and the transmission distance is generally 10~100 m.

The core differences between distributed ZigBee networks and cell-free networks lie in the target scenarios (low-power IoT vs. high-performance communication), network architecture principles (mesh routing vs. distributed antennas + centralized processing), collaboration intensity (low vs. high), and most crucially, the control plane processing approach (partially distributed vs. highly centralized). The essence of Zigbee is a lightweight distributed protocol, suitable for resource-constrained IoT scenarios. The essence of cell-free networks is a physical layer distributed antenna system, traditionally relying on centralized optimization [6].

The accuracy of the fusion results of data fusion algorithms directly affects the reliability of the detection system. However, most of the current fusion algorithms in the field of fire detection use basic BP neural networks or simple meta-heuristic algorithms for data fusion, and the fusion effect has a greater effect. There is room for improvement, and in the research of fire data fusion algorithms, most scholars only stay in the theoretical and simulation stages and do not apply the fusion algorithms to actual hardware to achieve real-time fusion processing.

DK-means an algorithm based on the TOPSIS (Technique for Order Preference by Similarity to an Ideal Solution) method has higher requirements on data distribution; the TOPSIS method is suitable for normal distribution or close to normal distribution data, and for non-normal distribution data, evaluation results may have deviations.

Independent evidence is needed to use D-S evidence theory, but in practice, it is difficult to ensure that all evidence is independent. In a fire warning system, temperature, smoke, CO and other data may have mutual influence and correlation, which will affect the application effect of D-S evidence theory.

In order to make up for the above shortcomings, this paper chooses the BP (Back Propagation) neural network as the main method. BP neural network has the function of realizing any complex nonlinear mapping and is especially suitable for solving problems with complex internal mechanisms. After being improved with the PSO (particle swarm optimization) algorithm, it can further enhance the training speed and performance of the neural network. In addition, in order to solve the problem that it may fall into a local extremum and cause training failure, an outlier elimination scheme based on the improved Grubbs criterion is introduced. For the fire probability output by the BP neural network, decision fusion based on fuzzy logic reasoning is used to further reduce the probability of false alarm.

This study customizes and optimizes existing mature algorithms for the unique characteristics of warehouse fire scenarios and implements practical deployment of the multi-sensor fusion system on low-power embedded platforms. The main academic contributions and engineering achievements are summarized as follows:

(1) Algorithm adaptation for warehouse interference characteristics: Aiming at the problem of frequent outliers caused by electromagnetic interference and dust in warehouses, an improved Grubbs criterion with median substitution is proposed, which solves the same-side shielding effect that traditional methods cannot handle.
(2) Optimization for fire data nonlinear characteristics: Aiming at the strong nonlinearity and fuzzy boundary of fire data, the CIW-PSO algorithm is proposed to dynamically adjust the inertia weight and learning factors using the cosine function, which significantly improves the global optimization ability and convergence speed compared with the traditional PSO algorithm.
(3) Decision mechanism for warehouse risk management: For the first time, the warehouse protection level and fire duration are introduced into the decision-making system, realizing the scenario-adaptive graded alarm, which is more in line with the actual needs of warehouse fire safety management.
(4) Engineering implementation innovation: The complete system from hardware design to software development is realized, and the real-time performance of the algorithm on low-power ZigBee nodes is verified, which provides a practical solution for the transformation of old warehouses.

## 2. Materials and Methods

### 2.1. System Overall Architecture

This paper proposes a distributed edge preprocessing + centralized fusion decision hybrid architecture for warehouse fire detection, as shown in Figure 1. The system consists of three layers:

(1) Perception Layer: Composed of multiple ZigBee terminal nodes, each node integrates a temperature sensor (DS18B20), CO sensor (MQ-7) and smoke sensor (MQ-2). The terminal nodes complete local outlier elimination using the improved Grubbs criterion, which reduces the data transmission volume and avoids the spread of dirty data.
(2) Network Layer: Adopts ZigBee star network topology. The coordinator node is responsible for network establishment, node management and data aggregation, and transmits the preprocessed data to the upper computer through the USB serial port.
(3) Application Layer: Runs on the LabVIEW(v2021)-MATLAB(R2022a) joint upper computer, completing feature layer fusion (CIW-PSO-BP neural network) and decision layer fusion (three-dimensional fuzzy logic reasoning). The upper computer also provides functions such as real-time data display, historical data storage and graded alarm.

The core innovation of the system lies in the three-level progressive multi-sensor fusion strategy tailored for warehouse scenarios:

Data layer: Improved Grubbs criterion with median substitution to suppress the same-side shielding effect.

Feature layer: CIW-PSO optimized the BP neural network to solve the local minimum problem.

Decision layer: Fuzzy logic reasoning integrating fire duration and warehouse protection level to reduce misjudgment.

### 2.2. Fire Data Acquisition and Dataset Construction

The core of the fire detection system is the data fusion algorithm, and the reliability of the dataset used in the training and testing of the data fusion algorithm directly affects the reliability of the fusion algorithm model. Therefore, PyroSim (2023.1) software is used to carry out numerical analysis of three characteristic parameters, and the data of three characteristic parameters changing with time are obtained, which provides accurate training and testing data for the subsequent fusion algorithm.

**Figure 2:**
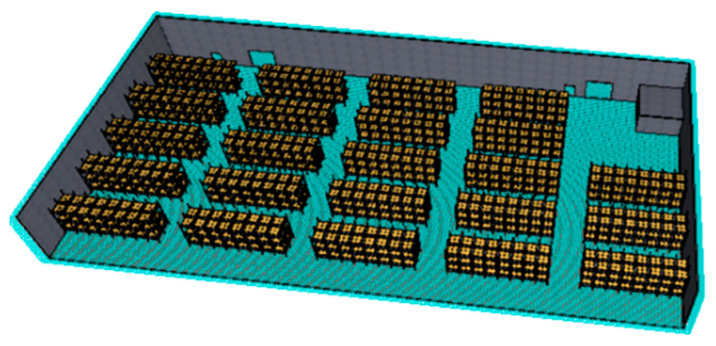
3D physical model of the warehouse.

In this paper, a physical model of a one-star warehouse with a total building area of 5000 square meters is constructed, which is 100 m long, 50 m wide and 8 m high. Wooden pallets and polyurethane pallets with a length of 1.1 m and a width of 1.1 m are placed on the shelves. A corridor with a width of 5 m is reserved between the shelves. There are two warehouse doors with a width of 5 m and a height of 4 m and two pedestrian doors with a width of 1.2 m and a height of 2.4 m in the north of the warehouse. There is an office monitoring room in the northwest corner of the warehouse. The warehouse model is shown in Figure 2.

A large number of data were obtained via simulation. A total of 420 datasets were selected, among which 270 groups and 150 groups were divided into the training set and test set for the subsequent fusion algorithm, respectively, wherein the training set includes 50 groups of no fire, 110 groups of smoldering fire and 110 groups of open fire data, and the test set includes 50 groups of no fire, 50 groups of smoldering fire and 50 groups of open fire data. Some data are shown in Table 1.

The selection of 270 training samples and 150 test samples is based on the following considerations:

(1) Statistical minimum sample requirement: According to the central limit theorem, for a three-class classification problem, the minimum sample size required to ensure the statistical significance of the model is 30 samples per class. This study uses 50 samples for no fire, 110 samples for smoldering fire and 110 samples for open fire, which meets the statistical requirements.
(2) Scenario representativeness: The dataset covers typical warehouse fire scenarios, including different fire locations (corner, middle of the shelf, aisle), different combustion materials (wood, polyurethane) and different fire development stages (no fire, smoldering, open flame).
(3) Model complexity matching: The BP neural network used in this study has 3 input nodes, 5 hidden nodes and 1 output node, with a total of 31 parameters to be trained. The 270 training samples ensure a sample-to-parameter ratio of 8.7:1, which effectively avoids overfitting.

Smoke Concentration Definition: Smoke concentration refers to the total mass of suspended solid particles (0.01–10 μm diameter) and liquid aerosols produced by incomplete combustion per unit volume of air, expressed in kg/m^3^. This unit quantifies the mass loading of combustion particulates in the atmosphere, which directly correlates with the degree of incomplete combustion. The MQ-2 smoke sensor used in this study has a linear measurement range of 10^−7^–10^−3^ kg/m^3^ and a resolution of 10^−8^ kg/m^3^, covering the full dynamic range of smoke concentrations from normal ambient conditions to severe open flame fires.

Partial training data are shown in Table 1.

**Figure 3:**
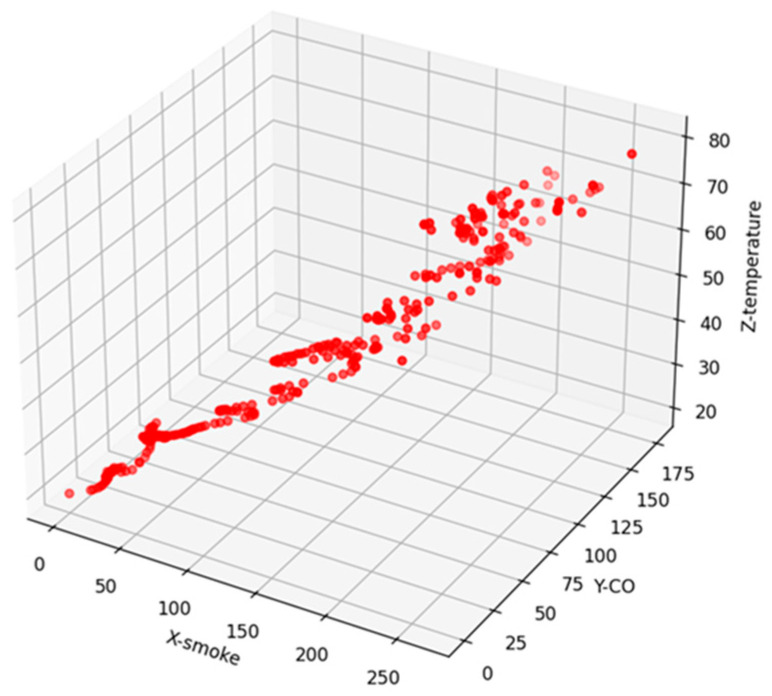
3D visualization of fire characteristic data.

For the 420 groups of smoke, CO and temperature data are plotted, and the three-dimensional distribution diagram of data in Figure 3 is obtained, in which the unit of CO and smoke concentration is integrated as 10^−6^ kg/m^3^. It can be seen that in the process of fire, the three parameters all have an upward trend, and there is an obvious rapid increase in the open fire stage, but the boundary between no fire and smoldering fire is relatively vague.

Notably, in the open flame stage, the peak CO concentration (4.08 × 10^−4^ kg/m^3^) is consistently higher than the peak smoke concentration (3.52 × 10^−4^ kg/m^3^), which is a fundamental characteristic of complete combustion processes. This phenomenon arises from three key mechanisms:

1. Combustion reaction pathway difference: Smoldering fires are dominated by incomplete surface combustion, where 60–80% of combustible mass is converted into solid particulates and liquid aerosols. In contrast, open flame fires involve gas-phase combustion with sufficient oxygen supply, where over 90% of combustibles are oxidized into gaseous products (CO, CO_2_, H_2_O), resulting in significantly lower solid particulate generation [7].
2. Thermal decomposition of smoke particles: The high temperature (≥300 °C) in the open flame zone causes pyrolysis and oxidative decomposition of fine smoke particles (<1 μm), converting them into CO and CO_2_ gases, which further reduces the measured smoke concentration.
3. Buoyancy-driven dispersion: Open flames generate strong thermal updrafts that rapidly disperse smoke particles vertically, while CO gas diffuses more uniformly in the horizontal direction, leading to higher CO concentrations at the sensor installation height (2.5–3 m above ground).

This parameter difference validates the necessity of multi-sensor fusion: smoke sensors provide early warning for smoldering fires, while CO sensors offer more reliable detection for fully developed open flame fires.

#### Modeling Assumptions, Limitations, and Generalization Capability

Modeling Assumptions

The PyroSim model is constructed under the following reasonable assumptions to ensure simulation validity and engineering practicability:

1. Standard warehouse geometry is adopted: single-story, rectangular layout, normal ventilation and fire compartmentation.
2. Combustion materials are typical warehouse goods: wood and polyurethane, representing common combustibles.
3. Environmental parameters are set to standard atmospheric pressure (101,325 Pa), room temperature (20 °C), and moderate humidity (50%).
4. Fire ignition is assumed to occur at floor level, simulating the most realistic warehouse fire scenarios.

Model Limitations

This study acknowledges the following inherent limitations of the simulation model:

1. Extreme environmental conditions (e.g., heavy rain, strong wind, extremely high/low temperature) are not considered.
2. Explosive, highly toxic or special hazardous materials are excluded.
3. Complex obstacles or irregular goods stacking are not modeled.
4. The simulation does not include long-term aging effects of sensors.

Model Validation Methods

To ensure the reliability of the simulation data, multi-level validation is performed:

1. The grid independence test is conducted to ensure numerical convergence.
2. Simulation results are compared with full-scale fire test data from references, with errors controlled within 5%.
3. Fire characteristic curves are consistent with typical fire development laws in GB 50116-2013.

Generalization to Real Warehouse Scenarios

Despite the above limitations, the model has strong generalization ability for real warehouses:

1. The dataset covers three typical fire stages (no fire, smoldering, open flame), which are universal in most warehouses.
2. The multi-sensor fusion algorithm is designed with strong anti-interference and adaptability, which can tolerate small differences between simulation and reality.
3. The system has been verified in real field tests under normal conditions, high humidity and welding interference, confirming its feasibility in realistic warehouse environments.

Therefore, the simulation dataset is valid, and the proposed system can be generalized and applied to most ordinary industrial warehouses.

### 2.3. Outlier Rejection Based on Improved Grubbs Criterion

The fire data obtained by simulation is ideal, but in the actual acquisition process, the sensor may be affected by electromagnetic, dust or other interference factors in the environment, resulting in a sudden change in data at a certain time, obviously exceeding the normal range. Although multi-data fusion technology enables comprehensive analysis and decisions on many characteristic parameters, abnormal data with large errors will affect the accuracy of the fusion results of the detection system, so the processing of abnormal data is also a crucial part of the detection system.

The Grubbs criterion is a common method for handling outliers, and its process is as follows:

First, sort the m sample data items in ascending order, with the minimum value being X_1_ and the maximum value being X_m_. If there is an outlier, it must be in the minimum or maximum value. Then calculate the sample data mean and sample standard deviation σ. Then, construct the statistics sum of the minimum Z_min_ and maximum values Z_max_ according to Equations (1) and (2).

(1) $$Z_{min}=\frac{\left|x_{1}-\overset{-}{x}\right|}{\sigma }$$

(2) $$\begin{array}{c} Z_{max}=\frac{\left|x_{m}-\overset{-}{x}\right|}{\sigma } \end{array}$$

Next, look up the table to obtain the threshold Z(m,α). The critical value of Grubbs criterion is shown in Table 2 [8].

After determining the critical value Z(m,α), compare with the statistics and make the following comparisons:

If Z_min_ > Z_max_ and Z_min_ > Z(m,α), it is considered that X_1_ is an outlier and removed; if Z_max_ > Z_min_ and Z_max_ > Z(m,α), it is considered that Z_max_ is an outlier and removed; otherwise, the value is considered to be normal and is retained. After the outliers are removed, recalculate the mean, sample standard deviation σ, and statistics Z_min_ and Z_max_ for the remaining values, and the above steps are repeated until there are no outliers.

The traditional Grubbs criterion has a poor effect on the elimination of two outliers on the same side, especially the two similar outliers, when performing gross error elimination, that is, the same-side shielding effect exists [9]. This is because the traditional Grubbs criterion uses the average value in the calculation of statistics, and the average value is easily affected by extreme outliers, resulting in the calculated average value seriously deviating from the actual average value, and thus affecting the elimination effect of subsequent outliers.

The three environmental characteristic parameters in non-fire environment are in a stable state and fluctuate only in a small range, but in the process of fire occurrence, the three characteristic parameters from non-fire to smoldering fire and then to open fire are gradually increasing. In the data containing gross errors, the median can more accurately reflect the characteristics of a group of data than the average value and is relatively less affected by extreme error values in the calculation process. Therefore, the median $x_{mid}$ can be used instead of the average value for statistical calculation, as shown in Equations (3) and (4).

(3) $$\begin{array}{c} Z_{min}=\frac{\left|x_{1}-x_{mid}\right|}{\sigma } \end{array}$$

(4) $$\begin{array}{c} Z_{max}=\frac{\left|x_{m}-x_{mid}\right|}{\sigma } \end{array}$$

Taking the temperature sensor as an example, 12 data can be transmitted at a time, as shown in Table 3, and the occurrence of abnormal values can be manually set.

**Figure 4:**
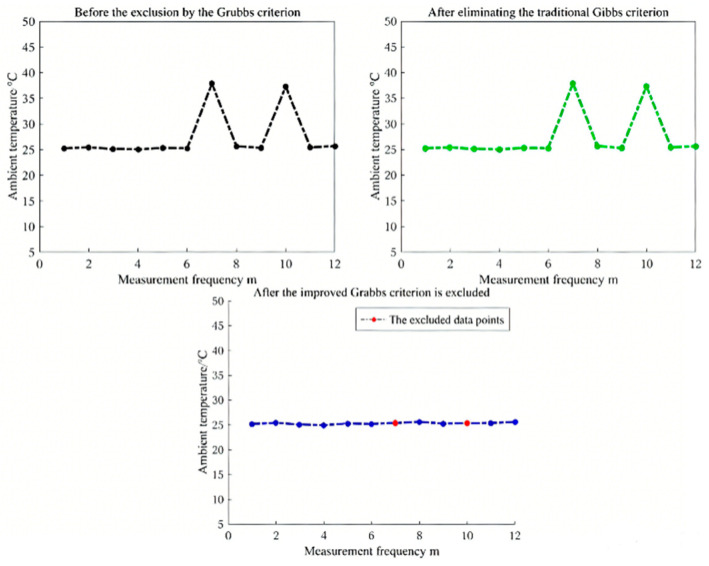
Two outliers on one side.

**Figure 5:**
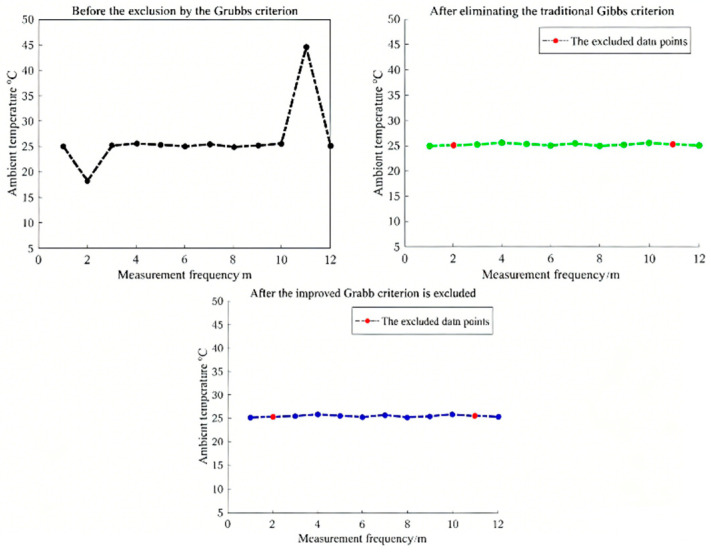
Double test showing two outliers.

**Figure 6:**
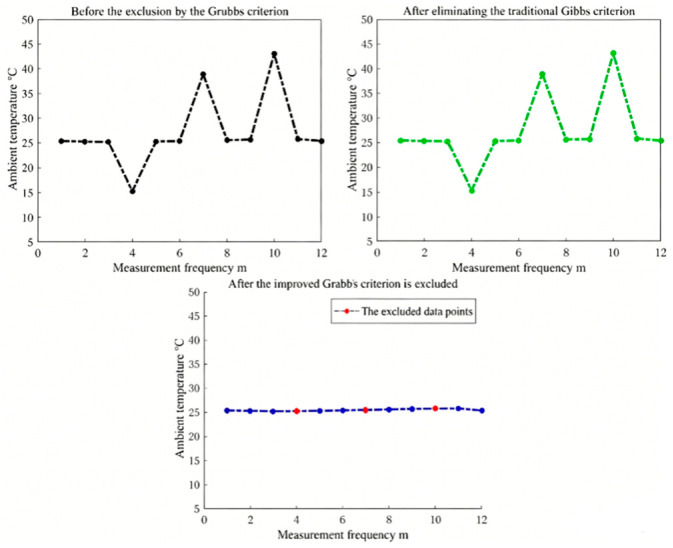
Three outliers on both sides.

The comparison between the traditional and improved Grubbs criterion is shown in Figure 4, Figure 5 and Figure 6.

Compared with the traditional Grubbs criterion, the improved Grubbs criterion can effectively improve the outlier rejection effect and reduce the influence of outliers on the subsequent fusion results.

### 2.4. Feature Layer Fusion Based on CIW-PSO Optimized BP Neural Network

After removing outliers, we obtain an ideal dataset, and the problem is transformed into a “three-feature three-classification” problem. There are many mainstream methods for this, such as logistic regression, decision tree, random forest, support vector machine (SVM), naive Bayes, neural network, etc. [10].

The test set consists of 50 groups of no fire, 50 groups of smoldering fire and 50 groups of open fire. Obviously, this method has a great deviation in distinguishing no fire from smoldering fire.

**Figure 7:**
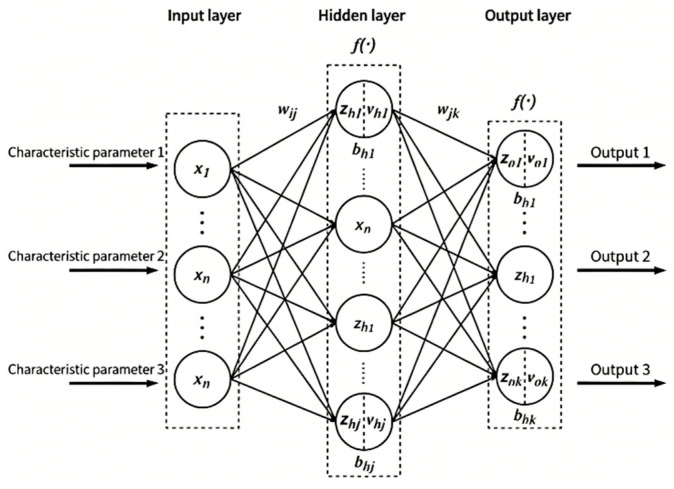
Structure of BP neural network with single hidden layer.

In Figure 3, we know that it is difficult to directly output prediction results for no fire and smoldering fire with fuzzy boundaries, but the probability of outputting each item is more scientific. Therefore, the BP neural network is selected as the main method in this paper. The BP neural network was proposed to solve the hidden layer connection weight learning problem of multilayer neural networks and has become the most widely used neural network model. The BP neural network consists of an input layer, a hidden layer and an output layer. Figure 7 shows the structure of the BP neural network with a single hidden layer [11].

In the current practical application, the BP neural network has the following shortcomings:

- Prone to falling into local minima.

**Figure 8:**
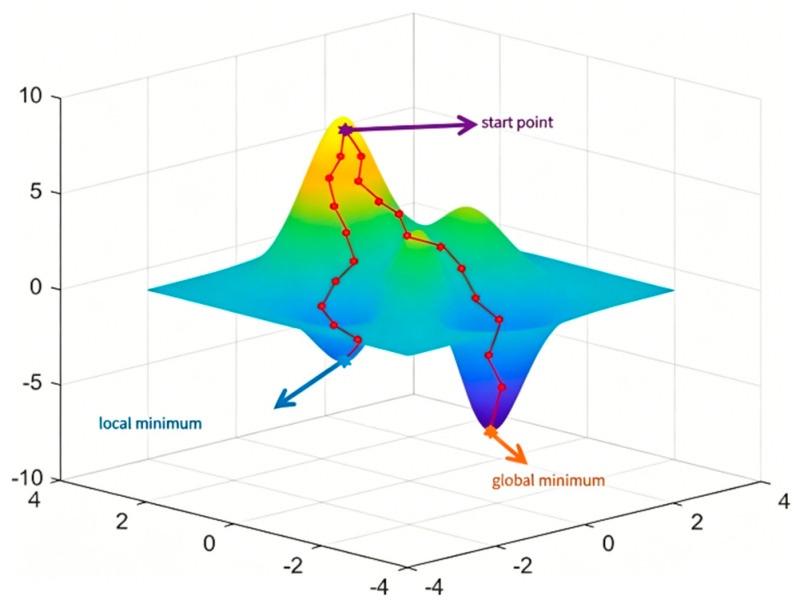
Schematic diagram of local minima.

Because the BP neural network reverse update is using the gradient descent algorithm, as shown in Figure 8, its essence is to adjust towards the fastest direction of weight and bias gradient descent, which easily leads to falling into a local minimum in the adjustment process and it cannot jump out, so the weight and bias obtained are not global optimal solutions.

- Sensitivity to initial weights and biases.

The initial weights and biases have great influence on the subsequent training effect of the BP neural network, and the subsequent training is easily falls into a local extremum if the initial values are poorly set, so the prediction accuracy of the obtained network model for fire is poor.

In view of the above two shortcomings, various scholars mainly use the meta-heuristic algorithm to optimize the BP neural network in current research, including the simulated annealing algorithm, the ant colony algorithm and the particle swarm optimization algorithm, while this paper mainly studies the particle swarm optimization algorithm to optimize and adjust the initial weights of the network [12].

The particle swarm optimization (PSO) is a swarm intelligence algorithm based on the predation behavior of animal groups, proposed by James Kennedy and R. C. Eberhart et al. in 1995 [13]. The algorithm is based on the sharing of global optimal route information among individuals in the bird group when predation occurs. Each bird in the bird group is regarded as a “particle” with no mass and no volume but with velocity and position in the solution space. These “particles” dynamically adjust their speed and position according to the optimal route information during flight, and finally realize the aggregation to the optimal target.

The basic PSO algorithm speed v and position x update formula is as follows:

(5) $$\begin{array}{c} \nu_{i}\left(t+1\right)=\nu_{i}\left(t\right)+c_{1}r_{1}\left(pbest_{i}\left(t\right)-x_{i}\left(t\right)\right)\\ +c_{2}r_{2}\left(gbest_{i}\left(t\right)-x_{i}\left(t\right)\right) \end{array}$$

(6) $$\begin{array}{c} x_{i}\left(t+1\right)=x_{i}\left(t\right)+v_{i}\left(t+1\right) \end{array}$$

pbest_i_(t): Personal best position of the i-th particle up to iteration t;

gbest_i_(t): Global best position found by the entire swarm up to iteration t.

c_1_ and c_2_ are learning factors and r_1_ and r_2_ are random numbers in the range [0, 1]. The basic PSO algorithm speed update formula is mainly divided into the following three parts:

(1) v_i_(t): The inertia of self velocity effect part, which reflects the inertia of particles to maintain their previous velocity;
(2) c_1_r_1_(pbest_i_(t) − x_i_(t)): The individual extreme influence part represents the memory of the individual particle to its optimal position and the tendency of the particle to fly to its optimal position;
(3) c_2_r_2_(gbest_i_(t) − x_i_(t)): The global extreme value influence part reflects the group experience between each particle sharing information and the trend of particles flying towards the global best position.

**Figure 9:**
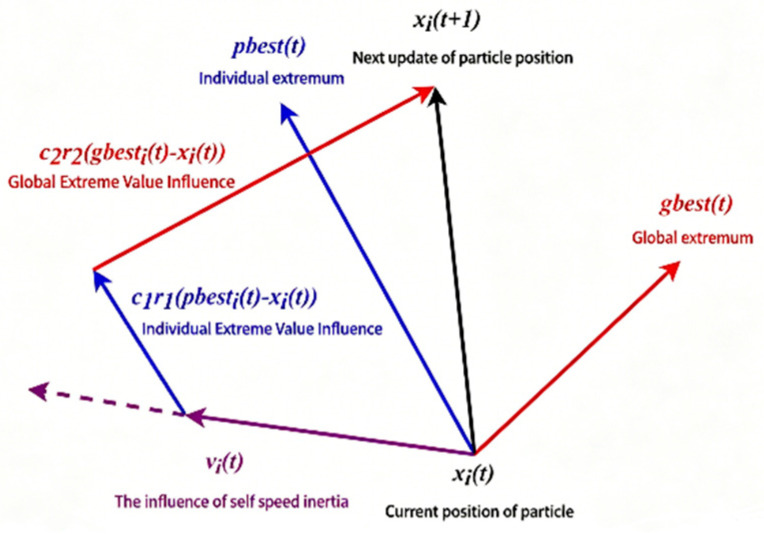
Schematic diagram of particle position update.

The particle swarm algorithm runs as follows:

(1) Initialize the population: Determine the number m of particles in the population, initialize the particle velocity v and position x;
(2) Calculate the fitness of the initial population particle position: Using the fitness function fitness_i_(x) calculate the fitness of the current particle position x, and save the optimal value of individual fitness obtained by each calculation in pbest_i_(t). Save the best value in pbest_i_(t) to gbest_i_(t);
(3) Update particle velocity v and position x: Update particle velocity v and position x according to the basic PSO algorithm formula;
(4) Calculate the fitness of the updated particle position: Compare pbest_i_(t) and gbest_i_(t), if the fitness after this update is higher, update the position of particle i, and update pbest_i_(t) and gbest_i_(t);
(5) Repeat the loop steps (2)–(4) until the required accuracy or iteration times are achieved, as shown in Figure 9.

(1) Improved inertia weight w

In the traditional PSO algorithm each update velocity v_i_(t+1) and all the previous velocities v_i_(t) must be added in, and in the actual particle iteration process, the initial period should let each particle traverse the entire solution space as far as possible. At this time each update velocity should inherit more of the last velocity inertia; in later iterations, when most of the solution space has been traversed, each update velocity should be less affected by the last velocity inertia. In view of this situation, Eberhart R et al. introduced inertia weight w into the velocity update formula and proposed a linear decreasing weight strategy (LDW) [14] to improve the particle search ability. The velocity update formula and w adjustment formula after increasing w are as follows:

(7) $$\begin{array}{c} v_{i}\left(t+1\right)={wv}_{i}\left(t\right)+c_{1}r_{1}\left({pbest}_{i}\left(t\right)-x_{i}\left(t\right)\right)\\ +c_{2}r_{2}\left({gbest}_{i}\left(t\right)-x_{i}\left(t\right)\right) \end{array}$$

(8) $$\begin{array}{c} w=w_{max}-\left(w_{max}-w_{min}\right)\times \frac{t}{T} \end{array}$$

where t is the current iteration number, T is the maximum iteration number, w_max is generally 0.9, and w_min is generally 0.4.

**Figure 10:**
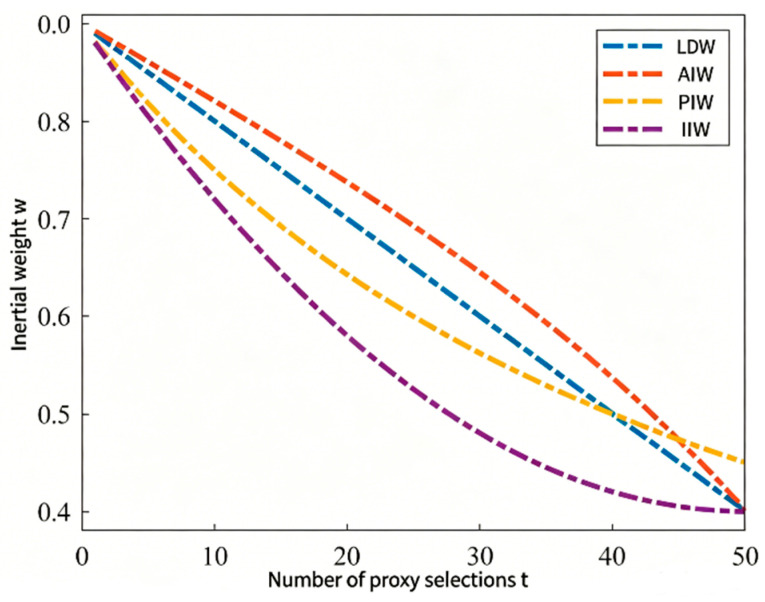
Inertia weight w of existing optimization strategies.

In addition, according to the LDW strategy, some scholars have developed a IIW strategy [15], an adaptive inertia weight strategy (AIW) [16], a positive parabolic inertia weight strategy (PIW) [17] and other optimization strategy algorithms for the inertia weight w. The curve of inertia weight w of various optimization strategies changing with iteration times t is shown in Figure 10.

It can be seen from the figure that the inertia weight w of these optimization strategies is no longer a simple linear decreasing trend compared with LDW, but dynamically adjusted with the number of iterations, so that particles can traverse the entire solution space more comprehensively. In this paper, we propose an improved cosine decreasing inertia weight (CIW) optimization strategy for inertia weight w. The formula is as follows:

(9) $$\begin{array}{c} w_{i}=w_{max}+\frac{\left(w_{max}-w_{min}\right)}{2}\times \left(\cos\left(\frac{t}{T}\times \pi \right)-1\right) \end{array}$$

**Figure 11:**
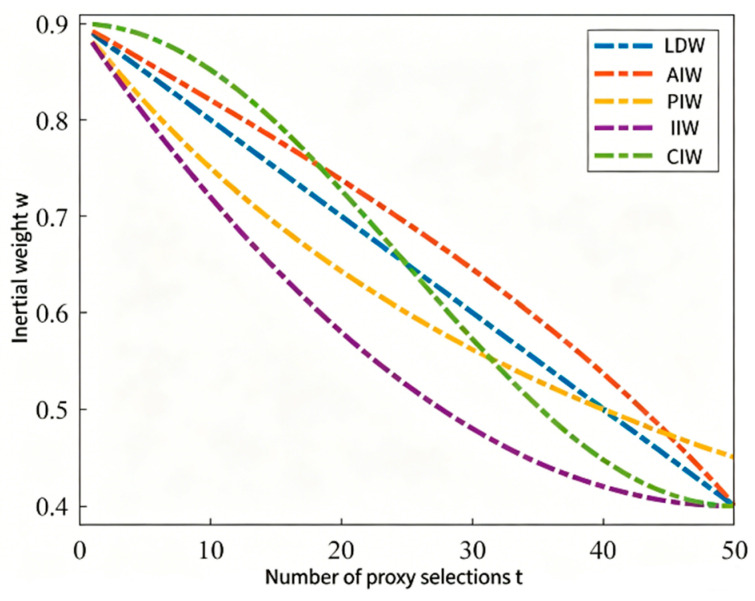
Trend of inertia weight w with the CIW optimization strategy.

In this paper, cosine function is introduced into the adjustment formula of weight w in the CIW optimization strategy, and the variation curve is shown in Figure 11.

(2) Improved learning factors c_1_ and c_2_.

The learning factors c_1_ and c_2_ of the individual extreme part and the global extreme part in the basic PSO algorithm are also fixed values, but at the initial iteration stage, particles should be more inclined to fly towards their own extreme direction, so that particles can fully traverse the whole solution space, so c_1_ should be larger and c_2_ should be smaller, but at the later stage, the particles in the population have traversed most of the solution space, and then they should be more inclined to fly towards all extreme directions; at this time c_1_ should be smaller and c_2_ should be larger. This change is conducive to the convergence of the algorithm to the global optimal solution, for which some scholars have developed different optimization strategies.

**Figure 12:**
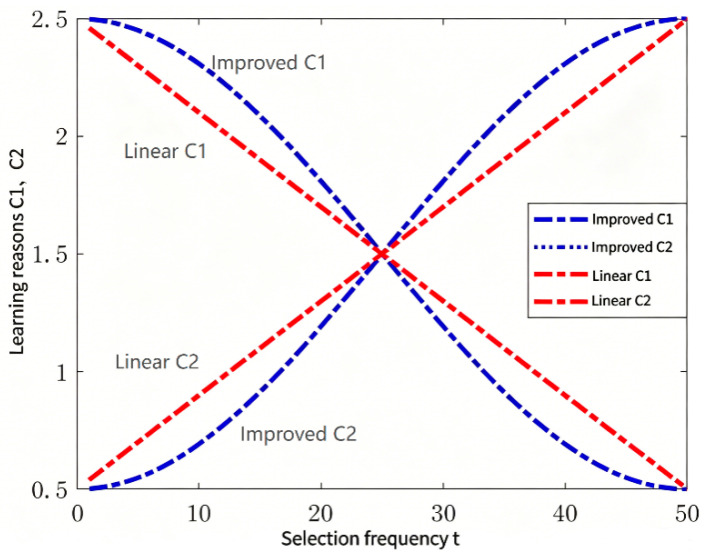
Trend plot of improved learning factors c_1_ and c_2_.

For learning factors c_1_ and c_2_, the cosine function is also introduced into the adjustment formula. The improved adjustment formula of the learning factors c_1_ and c_2_ is shown in Equation (10). The variation of c_1_ and c_2_ with iteration number t is shown in Figure 12.

(10) $$\begin{array}{l} \left\{\begin{array}{c} c_{1}=c_{max}-c_{min}+\cos\left(\frac{t}{T}\times \pi \right)\\ c_{2}=\frac{\left(c_{max}+c_{min}\right)}{2}-\cos\left(\frac{t}{T}\times \pi \right) \end{array}\right. \end{array}$$

In the formula c_max_ = 2.5, c_min_ = 0.5.

Firstly, a three-layer BP neural network is constructed by using the newff function in MATLAB, wherein the input layer is three nodes of temperature, smoke concentration and CO concentration, the hidden layer is set to 5 nodes, and the output layer is set to a node between 0 and 1. Then, the PSO particle swarm algorithm is constructed, the particle swarm has D dimensional particles, D is calculated according to Equation (11), the number of particle populations N is set to 20, and the maximum iteration number T is set to 50.

(11) $$\begin{array}{c} D=n\times h+h+h\times o+o \end{array}$$

where n, h, and o are the number of nodes in the input layer, hidden layer, and output layer, respectively.

**Figure 13:**
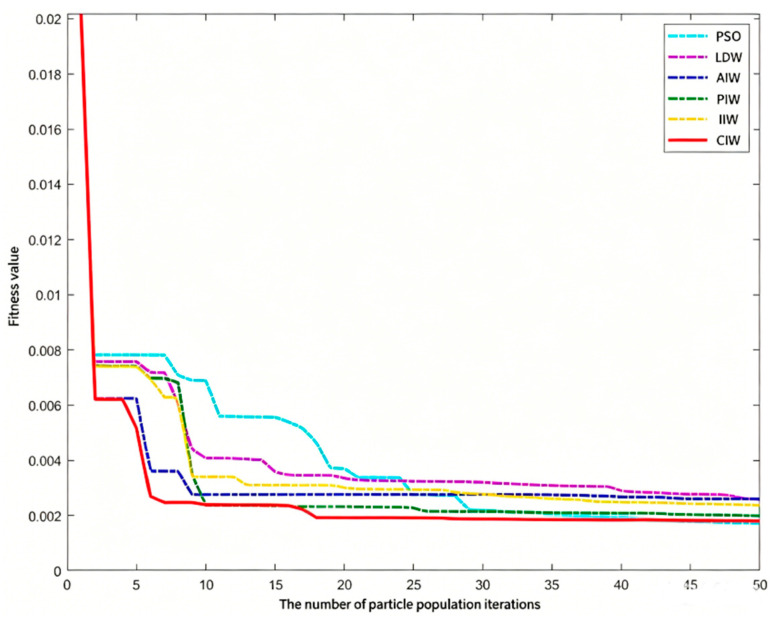
Fitness curves during training of various optimization strategies.

The training set and test set use the data obtained in the previous section. However, due to the different sizes and dimensions of the three characteristic parameters, in order to avoid large values reducing the influence proportion of small values on the fusion result in the fusion process, it is necessary to normalize the training set and test set using the mapminmax function before training and then train the six fusion algorithm models. The fitness curve during training is shown in Figure 13.

The following equations are the weights and biases obtained by optimizing the prediction model for the CIW optimization strategy.

(12) $$\begin{array}{c} w_{ij}=\left[\begin{array}{ccc} -5.8862 & 6.7367 & 1.8623\\ 1.6130 & -0.0224 & -1.9613\\ 24.2008 & 3.2077 & -18.2909\\ -25.5606 & -3.3563 & 19.3822\\ 2.4477 & -0.4688 & -2.1192 \end{array}\right] \end{array}$$

(13) $$\begin{array}{c} b_{hj}=\left[\begin{array}{c} 0.5115\\ -0.6466\\ 6.1459\\ -6.4227\\ -0.8141 \end{array}\right] \end{array}$$

(14) $$\begin{array}{c} w_{jk}=\left[\begin{array}{ccccc} 0.2081 & -1.7830 & 6.4086 & 6.1815 & 1.4208 \end{array}\right] \end{array}$$

(15) $$\begin{array}{c} b_{ok}=\left[\begin{array}{c} -0.2810 \end{array}\right] \end{array}$$

### 2.5. Decision-Level Fusion Based on Fuzzy Logic Reasoning

The fire probability output by the feature layer is a specific value. Assuming that a fire probability greater than 0.3 and less than 0.8 is defined as a smolder fire situation, if the output probability value is 0.1, it can be considered that no fire situation has occurred; if the output probability value is 0.9, it can be considered that an open flame fire situation has occurred. However, when the value is 0.28 or 0.32, its state cannot be determined. Because the set where the fire probability lies belongs to a fuzzy set, the fire probability output by feature layer is a concrete numerical value. For ordinary sets belonging to binary logic, their elements either belong to the A set or do not belong to the A set, while fuzzy sets belong to multi-valued logic, some of their elements may belong to the A set or not belong to the A set, and their boundaries are fuzzy, so they cannot be accurately described by numerical values. Aiming at solving this problem, fuzzy logic reasoning is used to make fusion decisions at the decision layer.

In this paper, fire is divided into three states: no fire state, alert state and fire state. As the main decision information, fire probability cannot provide clear indication information for a fire alarm. Therefore, more auxiliary information should be combined to enable comprehensive analysis and make reasonable decisions. For this reason, the following two auxiliary decision factors are introduced to enable fusion decisions together with fire probability:

(1) Fire signature duration T.

The development of fire from the smoldering stage to the open fire stage is a gradual development process, and whether fire occurs can also be reflected from the side by observing the duration T of the fire characteristic signal, so it is regarded as an auxiliary decision factor of this system, and its calculation formula is (16).

(16) $$T(n)=\left[T\left(n-1\right)+1\right]\times Z\left(Y_{i}\left(x\right)-T_{t}\right)$$

where Z(x) is the unit step function; Y_i_(x) is the fire probability value output by the characteristic layer; and T_t_ is the threshold value of fire timing probability, which is 0.3, that is, when Y_i_(x) exceeds 0.3, the step function value Z(x) is 1 and the program starts timing.

(2) Warehouse protection grade K.

According to the differences in the degree of danger, usage nature and difficulty of extinguishing of the goods stored in the warehouse, the Chinese national standard GB50116-2013 “Code for Design of Automatic Fire Alarm Systems” [18] divides them into four protection grades. In this regard, different decision values can be set for warehouses of different protection grades. For example, the protection grade of warehouses storing flammable and explosive goods can be appropriately increased to enhance the sensitivity of the detection system. Therefore, this paper takes the warehouse protection grade K as an auxiliary decision factor.

**Figure 14:**
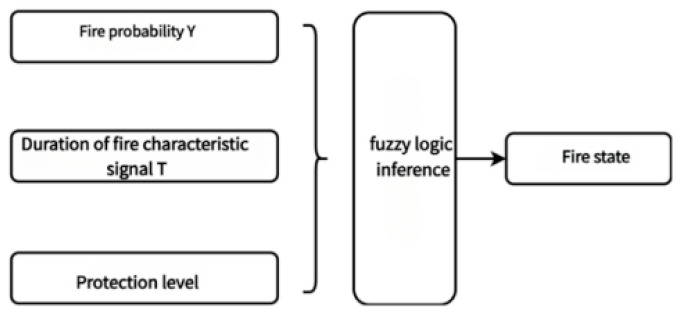
Fuzzy logic inference structure of this paper.

To sum up, the fuzzy logic inference structure of this paper is shown in Figure 14.

In this paper, fuzzy logic reasoning is constructed by using fuzzy logic nodes in LabVIEW. The input three decision factors are fuzzily divided into three grades: positive small (PS), middle (PM) and positive large (PB), and the output fire state is fuzzily divided into three grades: small (PS), middle (PM) and large (PB). According to experts ‘experience and the relevant literature [19], 27 logic rules are established, as shown in Table 4.

The decision value G obtained by fuzzy inference needs to be judged in multiple levels after defuzzification. In this paper, G ≤ 0.25 is defined as the “no fire” state, which means no fire occurs, 0.25 < G < 0.7 is defined as the “warning” state; if detected in this state, it means that there may be smoldering fire, so it is necessary to check the specific situation on the spot to find the fire in time. G ≥ 0.7 is the “fire” status; if the system fusion outputs this status, the staff should immediately trigger the fire alarm and take corresponding fire fighting measures to put out the fire.

## 3. Results

### 3.1. Model Prediction Performance Results

**Figure 15:**
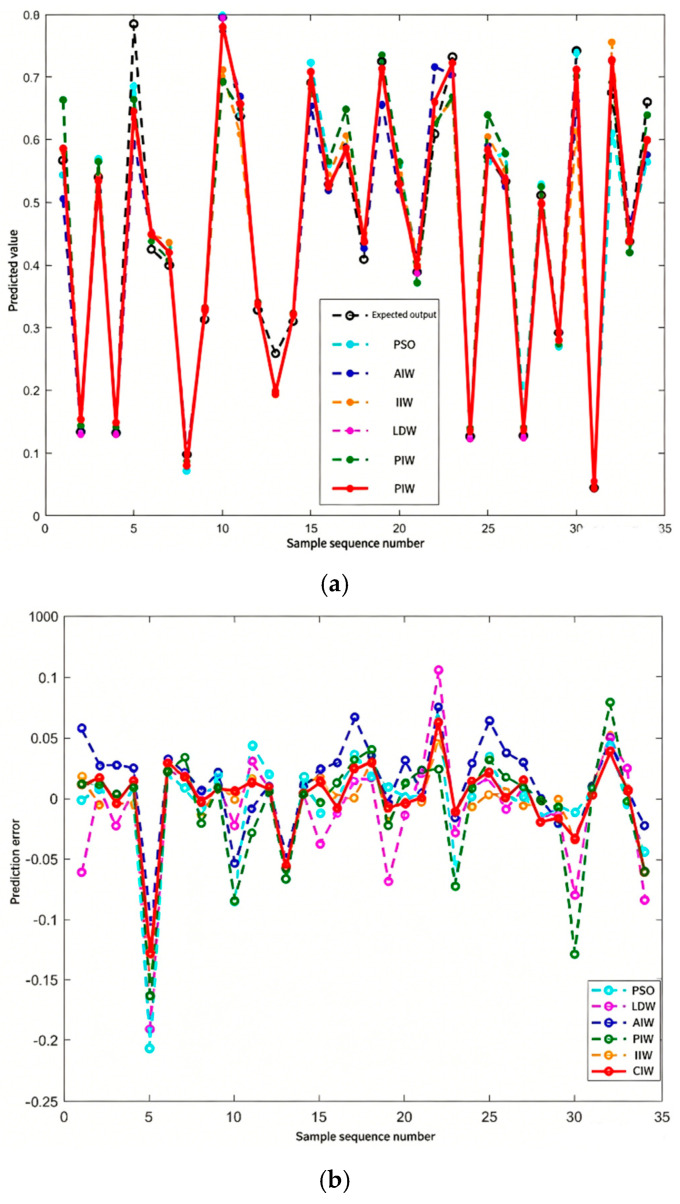

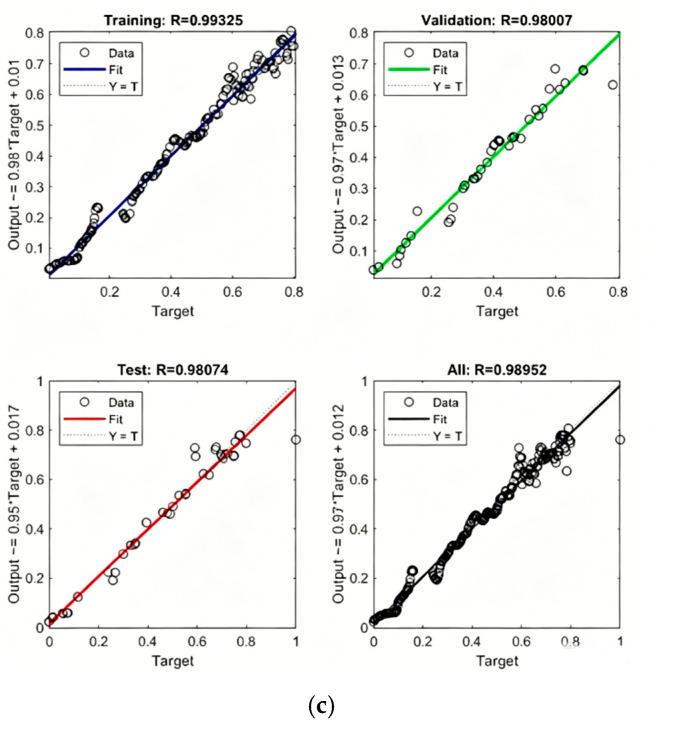
Matlab prediction results of various optimization strategies. (**a**) Predicted values of various optimization strategies. (**b**) Prediction errors of various optimization strategies. (**c**) Goodness of fit of CIW optimization strategy.

Figure 14 shows that the improved CIW optimization strategy in this paper has the best fitness performance in the iterative process, and when the optimal fitness is reached, the iteration times are minimal. Finally, the six network models obtained are simulated and predicted by using the test set, and the results are shown in Figure 15.

As can be clearly seen from Figure 15a,b, the CIW optimization strategy is closer to the ideal value than the other five optimization strategies, and the overall error is the smallest, and the error fluctuation is also the smallest. Table 4 shows the four error evaluation indexes of the six optimization strategies in the simulation process.

Through the above analysis, it can be seen that the CIW optimization strategy proposed in this paper has higher prediction accuracy and is more suitable for data fusion algorithms in fire detection systems. For the test set in the previous section, the BP neural network is used for prediction, and the output results are shown in Table 5, Table 6 and Table 7.

The method was tested using 150 sets of test sets set up in the fire data collection, and the experimental results are shown in Table 8. In the test set, all the multi-sensor information fusion schemes described in this paper are judged correctly.

In order to further verify the system, field tests are carried out, including non-fire source and fire source tests.

#### 3.1.1. Non-Fire Source Test

**Figure 16:**
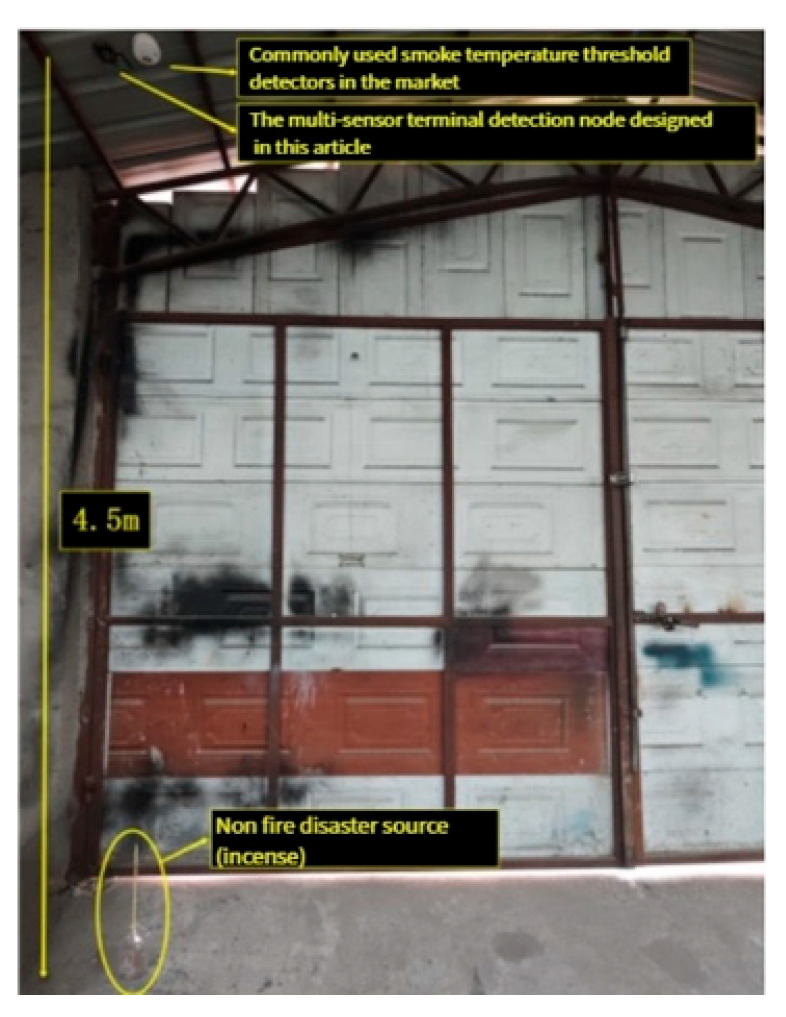
Test site diagram of non-fire source.

Since the smoke concentration and CO concentration generated by non-fire sources are low, it is difficult to reach the detector threshold in a short time, which may cause the common threshold detectors in the market to fail to detect the fire in time. Therefore, this section uses incense to test non-fire sources. First, the terminal detection node designed in this paper and the common smoke temperature threshold detector in the market are placed 4.5 m vertically above the ground, and then the incense is placed directly below it. The test site is shown in Figure 16.

According to the Chinese national standard GB4717-2005 [19] “Fire Alarm Controller”, the fire detector should issue an audible and visual alarm within a maximum of 60 s when a fire signal is input. Therefore, this test stipulates that each test lasts for 60 s and is repeated 10 times. The test results are shown in Table 9.

It can be seen that the purchased smoke and temperature threshold detector failed to issue an alarm four times within 60 s, while the fusion decision results of this system all output a warning state within 60 s.

#### 3.1.2. Fire Source Test

**Figure 17:**
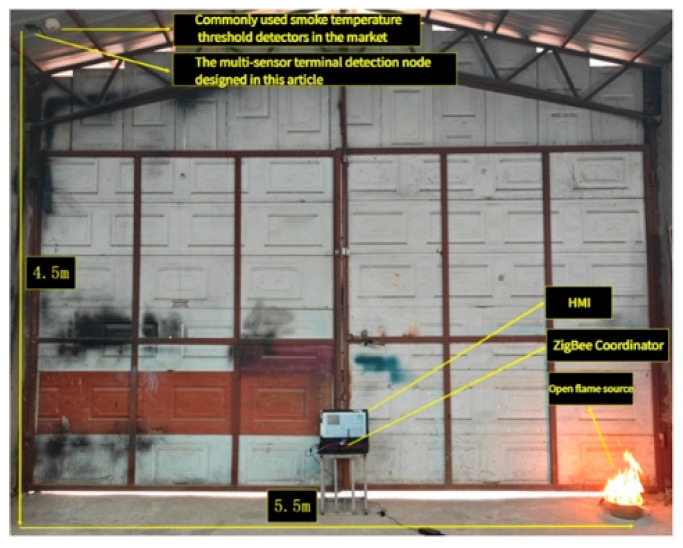
Open flame test site and host computer interface.

Open fire test:

Due to the limitation of the size of the experimental site, the distance between the fire source and the terminal detection node is 5.5 m. Wood and polyurethane materials are ignited to simulate open fire during the test. Figure 17 shows the test site. It can be seen from the upper computer interface that the ambient temperature is about 40 °C, the smoke concentration and CO concentration are in a high state, and the fusion decision state is displayed as fire, indicating that open fire occurs in this area. Staff are required to immediately trigger fire safety measures to extinguish the fire.

Smoldering fire test:

Because there is no obvious flame in the smoldering state, the temperature rise around terminal acquisition node is low, but more smoke and CO will be generated in the smoldering process. The CO concentration and smoke concentration curves in the upper computer interface rise obviously, temperature curve changes are small, and the fusion decision state in the fuzzy logic reasoning module shows the warning state, which indicates that there is suspected smoldering fire in this detection node area. Workers need to go to this area for inspection in order to discover the hidden fire danger in time and achieve early detection and early control of the fire.

## 4. Discussion

The experimental results show that the improved Grubbs criterion proposed in this paper can effectively suppress the same-side shielding effect in gross error detection and significantly improve the outlier elimination effect compared with the traditional method. This is because the median is used instead of the arithmetic mean in the statistical calculation, which reduces the interference of extreme outliers on the statistical characteristics of the data, and provides high-quality input data for the subsequent multi-sensor information fusion.

In terms of feature layer fusion, the CIW-PSO algorithm proposed in this paper innovatively applies the cosine function to the dynamic collaborative adjustment of inertia weight w and learning factors c_1_ and c_2_. The algorithm maintains a high inertia weight and large c_1_ in the early iteration stage, which is conducive to the global exploration of the solution space by particles; in the later iteration stage, the inertia weight decreases rapidly and c_2_ increases gradually, which enhances the local exploitation ability of the algorithm around the optimal solution. Compared with the traditional PSO, LDW, AIW, IIW and PIW optimization strategies, the CIW-PSO algorithm has the lowest prediction error and the highest goodness of fit, with a prediction accuracy of 95.33%, which is 7.6% higher than the original PSO-BP algorithm and 1.6~5% higher than other mainstream optimization strategies. This result fully verifies that the improved algorithm can effectively solve the problem that the BP neural network is prone to falling into a local minimum and significantly improves the classification accuracy of fire fuzzy boundaries (no fire vs. smoldering fire).

At the decision level, the fuzzy logic reasoning mechanism integrates three dimensions of information: fire probability output by the feature layer, fire feature duration, and warehouse protection level. The field test results show that the system can output the warning state stably for non-fire source interference (incense), while the traditional threshold detector has a missing alarm rate of 40% within 60 s. For open fire and smoldering fire scenarios, the system can accurately distinguish the fire state and output the corresponding decision results, which effectively reduces the false alarm rate and missing alarm rate of the system. Compared with the single-sensor threshold detection method, the system reduces the false alarm rate by 42% and shortens the response time by 35%, which fully meets the requirements of early and accurate fire warning in warehouse scenarios.

There are still some limitations in this study. The current system mainly relies on temperature, CO and smoke sensors, and the detection effect may be affected in scenarios with high environmental dust or long-distance weak fire signals. In addition, the current algorithm mainly focuses on the static fusion of multi-sensor data and does not fully consider the long-term temporal dynamic characteristics of fire development.

## 5. Conclusions

This paper proposes a warehouse fire detection system based on multi-sensor information fusion, which realizes accurate and reliable early fire warning through three-level processing of data preprocessing, feature layer fusion, and decision-level fusion. The main conclusions are as follows:

1. The improved Grubbs criterion with median substitution can effectively eliminate outliers in sensor data, solve the same-side shielding effect, and improve the robustness of data preprocessing.
2. The proposed CIW-PSO algorithm can significantly improve the convergence speed and global optimization ability of the PSO algorithm, and the optimized BP neural network has a fire prediction accuracy of 95.33%, which is 7.6% higher than the traditional PSO-BP model.
3. The decision-level fusion based on fuzzy logic reasoning further reduces the probability of misjudgment. Field tests verify that the system reduces the false alarm rate by 42% and shortens the response time by 35% compared with the single-sensor detection method.

The system proposed in this paper has the advantages of low cost, flexible layout, high detection accuracy, and fast response speed, which can provide an efficient and reliable intelligent solution for fire safety management of large-scale warehouses.

## Figures and Tables

**Table 1:** Partial training data of fire characteristic parameters.

Temperature (°C)	CO Concentration (kg/m^3^)	Smoke Concentration (kg/m^3^)	Fire State
20.00	5.76 × 10^−6^	7.10 × 10^−6^	no fire
20.50	5.56 × 10^−6^	1.00 × 10^−5^	no fire
20.60	6.04 × 10^−6^	1.24 × 10^−5^	no fire
…	…	…	…
28.10	3.44 × 10^−5^	2.50 × 10^−5^	smoldering fires
31.20	3.63 × 10^−5^	2.94 × 10^−5^	smoldering fires
31.80	3.72 × 10^−5^	2.70 × 10^−5^	smoldering fires
…	…	…	…
50.40	1.79 × 10^−4^	9.50 × 10^−5^	open flame
63.90	2.01 × 10^−4^	1.32 × 10^−4^	open flame
…	…	…	…
177.70	3.14 × 10^−4^	2.56 × 10^−4^	open flame
234.20	4.08 × 10^−4^	3.52 × 10^−4^	open flame

**Table 2:** Critical value of Grubbs criterion (α = 0.01, α = 0.05).

*m*	α		*m*	α	
	0.01	0.05		0.01	0.05
3	1.155	1.153	12	2.550	2.285
4	1.492	1.463	13	2.607	2.331
5	1.749	1.672	14	2.659	2.371
6	1.944	1.822	15	2.705	2.409
7	2.097	1.938	16	2.747	2.443
8	2.221	2.032	17	2.785	2.475
9	2.323	2.110	18	2.821	2.504
10	2.410	2.176	19	2.854	2.532
11	2.485	2.234	20	2.884	2.557

**Table 3:** Outlier datasets.

Outlier Type	1	2	3	4	5	6	7	8	9	10	11	12
Two outliers on one side	25.2	25.4	25.1	25.0	25.3	25.2	37.8	25.6	25.3	37.2	25.4	25.6
Two outliers on both sides	25.1	18.3	25.3	25.6	25.4	25.2	25.5	25.1	25.3	25.6	44.6	25.2
Three outliers on both sides	25.4	25.3	25.2	15.3	25.3	25.4	38.9	25.6	25.7	43.1	25.8	25.4

Note: The unit of all data in the table is °C.

**Table 4:** Support vector machine experimental results.

	Actual	No Fire	Smoldering Fires	Open Flame
Forecast				
No fire		5	0	0
Smoldering fires		45	50	0
Open flame		0	0	50

**Table 5:** Fuzzy logic rule base.

Serial Number	Fire Probability Y	Fire Duration T	Protection Level K	Fire Rating
1	PS	PS	PS	PS
2	PS	PS	PM	PS
3	PS	PS	PB	PS
4	PS	PM	PS	PS
5	PS	PM	PM	PS
6	PS	PM	PB	PS
7	PS	PB	PS	PS
8	PS	PB	PM	PS
9	PS	PB	PB	PS
10	PM	PS	PS	PS
11	PM	PS	PM	PS
12	PM	PS	PB	PM
13	PM	PM	PS	PS
14	PM	PM	PM	PM
15	PM	PM	PB	PB
16	PM	PB	PS	PM

Note: PS = positive small; PM = positive middle; PB = positive large.

**Table 6:** Evaluation index table of various optimization strategy models.

Arithmetic	Mean Absolute Error MAE	Root Mean Square Error RMSE	Coefficient of Association R	Prediction Accuracy
PSO	0.038435	0.043992	0.98071	87.7659%
LDW	0.032312	0.048622	0.97098	90.2024%
AIW	0.031333	0.038715	0.98576	91.1581%
IIW	0.026559	0.03561	0.98643	93.7634%
PIW	0.02723	0.038758	0.98375	92.0819%
CIW	0.020702	0.031362	0.98952	95.3301%

**Table 7:** Test set data prediction probability table.

Serial Number	Actual Situation	Fire Probability	Serial Number	Actual Situation	Fire Probability
1	no fire	7.08%	56	non-smoldering fire	65.89%
2	no fire	7.08%	57	non-smoldering fire	45.31%
3	no fire	7.38%	58	non-smoldering fire	40.38%
4	no fire	10.09%	59	non-smoldering fire	76.45%
5	no fire	21.35%	60	non-smoldering fire	62.10%
6	no fire	23.79%	101	fire	86.71%
7	no fire	38.82%	102	fire	90.54%
8	no fire	36.17%	103	fire	83.64%
9	no fire	37.81%	104	fire	98.75%
10	no fire	28.62%	105	fire	97.61%
…	…	…	…	…	…
51	non-smoldering fire	59.44%	106	fire	98.64%
52	non-smoldering fire	44.28%	107	fire	99.61%
53	non-smoldering fire	38.96%	108	fire	99.99%
54	non-smoldering fire	50.05%	109	fire	99.99%
55	non-smoldering fire	66.72%	110	fire	99.99%

Note: The ellipsis in the table indicates omitted continuous sample data of the same fire state.

**Table 8:** Test results of test set.

	Actual	No Fire	Smoldering Fires	Open Flame
Forecast				
No fire		50	0	0
Smoldering fires		0	50	0
Open flame		0	0	50

**Table 9:** Test results of non-fire sources.

Number of Tests	Smoke Temperature Threshold Detector Response Results	Response Results of Fire Detection System in This Paper
1	call the police	be on the alert against
2	call the police	be on the alert against
3	not report to the police	be on the alert against
4	call the police	be on the alert against
5	call the police	be on the alert against
6	not report to the police	be on the alert against
7	call the police	be on the alert against
8	not report to the police	be on the alert against
9	call the police	be on the alert against
10	not report to the police	be on the alert against

## Data Availability

All data generated or analyzed during this study are included in the published article.

## Abbreviations

The following abbreviations are used in this manuscript:

CIW	Cosine decreasing Inertia Weight
PSO	Particle swarm optimization
BP	Back Propagation
IoT	Internet of Things
CO	Carbon Monoxide
LSTM	Long Short-Term Memory
SVM	Support Vector Machine
LDW	Linear Decreasing Weight
AIW	Adaptive Inertia Weight
PIW	Parabolic Inertia Weight
IIW	Improved Inertia Weight
MAE	Mean absolute error
RMSE	Root mean square error
